# Food Insecurity, Physical Activity, and Sedentary Behavior in Middle to Older Adults

**DOI:** 10.3390/nu17061011

**Published:** 2025-03-13

**Authors:** Shiliang Chen, Zhiyong Li, Yanjie Zhang, Shihui Chen, Wenjiao Li

**Affiliations:** 1 School of Liberal Arts, Shenzhen City Polytechnic, Shenzhen 518116, China; slchensz@163.com (S.C.); 13751153496@163.com (Z.L.); 2 Physical Education Unit, School of Humanities and Social Science, The Chinese University of Hong Kong, Shenzhen 518172, China; zhangyanjie@cuhk.edu.cn; 3 Department of Kinesiology, Texas A&M University, Texarkana, TX 75503, USA; schen@tamut.edu

**Keywords:** physical activity, sedentary behavior, food insecurity, middle and older adults

## Abstract

**Purpose:** The present study investigates the associations between food insecurity, physical activity, and sedentary behavior in individuals aged 55 years and older from five low- and middle-income countries (LMICs). **Methods:** The data were provided by 16,454 adults aged 55 and older who participated in the Global Aging and Adult Health Survey. Food insecurity was assessed based on participants’ self-reported questionnaire measures. Physical activity and sedentary behavior were assessed using the Global Physical Activity Questionnaire, and adherence to the recommended guidelines was categorized accordingly. We used a multivariable logistic regression model to examine the associations between food insecurity, physical activity, and sedentary behavior, adjusting for demographic and lifestyle factors. **Results:** The results revealed that food insecurity was significantly associated with a lower likelihood of meeting physical activity (OR = 0.73, 95% CI: 0.64–0.83) and sedentary behavior recommendations (OR = 0.70, 95% CI: 0.59–0.83). Stratified analysis showed that food insecurity had a stronger impact on these behaviors among females than males. Females with food insecurity were less likely to meet all movement behavior recommendations, highlighting gender-specific challenges. **Conclusions:** The findings showed that food insecurity was significantly associated with lower adherence to both physical activity and sedentary behavior recommendations among middle-aged and older adults in LMICs. In these settings, females with food insecurity showed more consistent and significant associations than males. Future longitudinal studies should be conducted to systematically examine whether an enhancement of food security causes continuous changes in movement behaviors.

## 1. Introduction

Food insecurity, characterized by the inability to obtain sufficient, safe, and nutritious food as a result of socioeconomic limitations [1,2], remains an urgent public health issue worldwide [1,2]. This issue not only influences basic survival but also perpetuates the cycle of poor health, with particularly severe impacts on low- and middle-income countries (LMICs) [3,4], where an underdeveloped economy, climate change, and escalating agricultural challenges further exacerbate vulnerability [5,6]. According to the 2019 United Nations report, it is estimated that approximately 1.3 billion people globally (17.2% of the population) lacked a reliable way to obtain adequate nutrition [7]. For example, South Africans experienced moderate to severe food insecurity in 2020, highlighting the dire situation faced by the region [8]. This issue ranks among the top five challenges faced by older individuals in South Africa, driving efforts to enhance healthcare accessibility and system responsiveness [1,9]. In Mexico, approximately 44.3% of the population encountered varying degrees of food insecurity, rendering around 49.9 million individuals vulnerable to this challenge [10]. In South Asia, countries such as India and Bangladesh face similar scenarios. Over 25% of the population in Bangladesh experienced moderate food insecurity in 2019, which was caused by poverty and agrarian distress [11]. These figures highlight a global emergency that is intertwined with public health and economic stability.

Households with food insecurity often adjust food budgets, reduce intake, and alter food types. This leads to a decrease in dietary diversity and an increased reliance on calorie-dense, nutrient-poor staples like refined grains, sugars, and unhealthy fats, which are cheaper per calorie [12,13]. For example, adults in households with food insecurity often eat fewer fruits, vegetables, and dairy products than their food-secure peers, leading to ab insufficient intake of micronutrients such as vitamins B and C, iron, phosphorus, zinc, and calcium [14,15]. This type of hidden hunger creates a vicious cycle: an insufficient intake of micronutrients can impair physical and cognitive functions, and it limits productivity and the income-generating capacity, thereby exacerbating poverty [2,16]. Even worse, irregular dietary patterns disrupt metabolic health and increase the risk of obesity [17], malnutrition [18], hypertension [19], heart disease [2], and other chronic diseases [12,20], along with mental health problems [21]. Previous studies have revealed that individuals with food insecurity are 40% more likely to develop chronic conditions such as diabetes and cardiovascular disease, even after adjusting for socioeconomic factors [22,23]. Other studies show that food insecurity is associated with a two- to threefold increase in the risk of depression and anxiety among adults [24,25].

In addition to affecting nutrient intake and chronic diseases, food insecurity may also result in changes in physical activity behaviors due to limited access to nutritious food or uncertainty in obtaining healthy food options [26]. Generally, individuals experiencing food insecurity often have reduced energy levels physically (e.g., fatigue and illness) and mentally (e.g., stress, depression, and anxiety), which can lead to a decreased probability of participating in physical activity and an augmented risk of adverse health outcomes. For instance, an observational study by Martinez et al. found that food insecurity was associated with fewer frequency of physical activity a weekamong college students [27]. Bruening et al. reported that among a sample of freshmen from Arizona, individuals with food insecurity were more prone to engage in less physically demanding forms of exercise compared to their peers without food insecurity [28]. To and colleagues found that food insecurity was significantly associated with a reduced probability of adhering to physical activity among a US population [29]. Regarding sedentary behavior, the existing literature has reached inconsistent conclusions. Some studies have reported various relationships (e.g., positive, negative, and null) between food insecurity and sedentary time [29,30]. For instance, one observational study found no correlation between food insecurity and sedentary behavior among adults [29]. Navarro et al. found that food insecurity was significantly associated with engaging in more sedentary behaviors among US youth [30]. Taken together, most of the current research focuses on young people or high-income countries. Young people experiencing food insecurity are unlikely to adhere to the World Health Organization’s guidelines on physical activity and sedentary behavior, which recommend 150 min of moderate-intensity or 75 min of vigorous-intensity physical activity weekly and less than 8 h of sedentary behavior a day [27,28,29,31,32,33]. However, the relationships between food insecurity and movement behaviors (e.g., sufficient physical activity and limited sedentary behavior) among middle-aged and older adults remain unclear.

As mentioned earlier, emerging studies have found food insecurity, insufficient physical activity, and sedentary behavior can significantly impact health outcomes [34,35,36,37]. Accordingly, these factors may become key targets for intervention strategies and policymaking. Therefore, the aim of the current study was to investigate whether food insecurity was related to physical activity and sedentary behavior among middle to older adults from LMICs.

## 2. Methods

### 2.1. Participants

Data for the present study were retrieved from the Global Ageing and Adult Health Survey (SAGE), conducted between 2007 and 2010 to monitor the health condition among middle to older adults in LMICs (e.g., China, India, Ghana, Russian Federation, and South Africa). At the time of data collection, Ghana was classified as a low-income country, while the remaining countries fell under the middle-income category based on the World Bank classification criteria. The methodological details of this survey have been comprehensively documented in earlier studies [38,39]. In brief, this survey employed a multi-stage sampling design to ensure a nationally representative sample. The sample consisted of individuals aged 18 years and greater, with an oversampling of adults aged 50 and older to understand aging-related health dynamics. Professionally trained staff provided in-person instructions to help participants accurately complete the standardized questionnaires during the data collection process. Response rates of this survey were 93% in China, 68% in India, 81% in Ghana, 83% in the Russian Federation, and 75% in South Africa. More details about SAGE have been published elsewhere [40]. This study adhered to the ethical guidelines outlined in the Declaration of Helsinki and was approved by the WHO Ethical Review Committee and local Human Sciences Research Council in each country [41].

For this study, we included in the sample to those who were at least 55 years old and excluded those missing data on demographics, physical activity, sedentary behavior, and food insecurity. Finally, 16,454 adults (≥55 years) with complete data on food insecurity, physical activity, sedentary behavior, and demographics were included for our analysis in this study.

### 2.2. Food Insecurity

Food insecurity was assessed using participants’ responses to two specific questions: “In the last 12 months, how often did you ever eat less than you felt you should because there wasn’t enough food?” and “In the last 12 months, were you ever hungry, but didn’t eat because you couldn’t afford enough food?” Both questions provided the following answer options: every month (=1); almost every month (=2); some months, but not every month (=3); only in one or two months (=4); never (=5). These questions are designed based on similar questions identified in food security questionnaires (e.g., the Household Food Security Survey Module in the United States and the Food Security Module of the National Health and Nutrition Examination Survey). Individuals who responded with 1 to 3 to both questions or 1 to either question were considered as food insecure. Thus, food insecurity was coded as 1 and food security was coded as 0 in our study. This approach mirrors methods in previous studies [42,43].

### 2.3. Physical Activity

The physical activity levels of participants were assessed using the Global Physical Activity Questionnaire (GPAQ) [44]. This tool assessed moderate and vigorous physical activity by recording the number of days per week and the duration per day (in hours and minutes) across three forms: occupational, transportation-related, and recreational activities. The total physical activity was calculated by combining these three forms and converting the results into metabolic equivalent tasks (METs) in minutes per week (MET-min). Based on MET-min values, physical activity was categorized into three levels: insufficient (0–600 MET-min weekly), moderate (601–3000 MET-min weekly), and vigorous (>3000 MET-min weekly) [45]. Achieving moderate to vigorous physical activity (MVPA) was considered as meeting the physical activity recommendation, and this was coded as 1 [46]. Conversely, it was coded as 0.

### 2.4. Sedentary Behavior

Daily sedentary behavior was assessed by asking participants one question, which was adapted from the GPAQ: “How much time do you usually spend sitting or reclining on a typical day? Here are examples: working at a desk, socializing while seated, commuting by car, bus, or train, reading, playing cards, or watching television” [47]. Sedentary behavior was analyzed in the present study using two methods: continuous and categorical measures. The continuous measure represented the total hours per day spent engaging in sedentary activities, and the categorical measure divided participants based on daily sedentary time. Those who reported fewer than 8 h per day were categorized as meeting the sedentary behavior recommendation (coded as 1), while those with 8 or more hours per day were classified as not meeting recommendations (coded as 0) [45].

### 2.5. Control Variables

Based on previous studies, control variables were selected [43,48]. The control variables consisted of age, gender, education level, marital status, residential area, number of chronic diseases (e.g., diabetes, hypertension, arthritis, and stroke), smoking (current, past, and never smoking), and alcohol consumption. In marital status, both marriage and cohabiting were coded as “cohabiting”, while others (such as unmarried, divorced, and widowed) were coded as “not cohabiting”. Diabetes, arthritis, and stroke were identified based on self-reported diagnoses. Hypertension was identified as either a self-reported diagnosis or measured blood pressure values of ≥140 mmHg systolic or ≥90 mmHg diastolic. The number of chronic diseases was quantified based on the aggregation of self-reported diagnosed conditions [48], classified as 0, 1–2, and ≥3. The education levels were categorized as less than elementary school (0), elementary school (1–6 years), middle school or high school (7–12 years), and college or post-graduate degree (>12 years).

### 2.6. Statistical Analysis

Stata 18.0 (Stata Corp LP, College Station, TX, USA) was used to perform all statistical analyses. Statistical significance was set at *p* < 0.05 (two-tailed). Descriptive statistics were employed to summarize the study sample. Means and standard deviations (SDs) were calculated for continuous variables, and frequencies along with percentages were generated for categorical variables. We used a multivariable logistic regression model to investigate the associations between food insecurity, physical activity, and sedentary behavior. The regression model was conducted by controlling for age, gender, education years, number of chronic diseases, smoking, and alcohol consumption. Moreover, another multivariable logistic regression model was conducted to stratify by gender and investigate the associations between food insecurity, physical activity, and sedentary behavior, while controlling for covariates. Odds rates (ORs) with 95% confidence intervals (CIs) were presented for each association.

## 3. Results

Table 1 summarizes the characteristics of the sample (*n* = 16,454), with a mean age of 65.47 ± 8.18 years. Nearly half of sample were male (50.75%). Approximately 44.34% of participants had 1–6 years of education, and 19.76% reported no formal education. Most participants (70.96%) were married, and the majority (60.86%) resided in urban areas. Over half (54.05%) reported alcohol consumption, while 25.96% were current smokers, 10.16% were past smokers, and 63.88% reported never smoking. Regarding chronic disease, 46.64% of individuals were afflicted with one to two chronic diseases and 13.86% of them had three or more. Furthermore, 7.35% of participants resided in households experiencing food insecurity. In regard to meeting movement behavioral recommendations, 88.19% met the sedentary behavior recommendation, 63.59% met the physical activity recommendation, and 58.44% adhered to both the physical activity and sedentary behavior recommendations.

### 3.1. Associations Between Food Insecurity, Physical Activity, and Sedentary Behavior

Table 2 presents the associations between food insecurity and adherence to physical activity and sedentary behavior recommendations. Participants with food insecurity were less likely to adherence to the physical activity recommendation (OR = 0.73, 95% CI: 0.64–0.83, *p* < 0.001), sedentary behavior recommendation (OR = 0.70, 95% CI: 0.59–0.83, *p* < 0.001), and both physical activity and sedentary behaviors recommendations (OR = 0.72, 95% CI: 0.63–0.81, *p* < 0.001) compared to those who did not have food insecurity.

### 3.2. Associations Between Food Insecurity, Physical Activity, and Sedentary Behavior Stratified by Sex

The sex-stratified analyses (Table 3) revealed that the relationships between food insecurity, physical activity, and sedentary behavior varied by gender. Among males, food insecurity was associated with a lower risk of meeting the sedentary behavior recommendation (OR = 0.61, 95% CI: 0.48–0.78, *p* < 0.001) and both physical activity and sedentary behavior recommendations (OR = 0.81, 95% CI: 0.68–0.96, *p* < 0.05). However, its association with meeting the physical activity recommendation was not statistically significant (OR = 0.88, 95% CI: 0.73–1.05, *p* = 0.15).

In females, food insecurity was significantly related to reduced odds of meeting all recommendations. Specifically, females who had food insecurity were less likely to adhere to the physical activity recommendation (OR = 0.61, 95% CI: 0.51–0.73, *p* < 0.001), the sedentary behavior recommendation (OR = 0.77, 95% CI: 0.61–0.98, *p* < 0.05), and both physical activity and sedentary behavior recommendations (OR = 0.63, 95% CI: 0.53–0.75, *p* < 0.001).

## 4. Discussion

The present study aimed to investigate the associations between food insecurity and adherence to physical activity and sedentary behavior recommendations, with additional analysis stratified by gender. The findings from our study showed that food insecurity was significantly related to reduced adherence to guidelines for physical activity, sedentary behavior, and both physical activity and sedentary behavior. Moreover, there were notable gender-based differences, whereby the association between food insecurity and health-related movement behaviors was more significant among females than males.

Consistent with previous studies reporting an association between food insecurity and physical activity among adults from a high-income country (US) [29,49] and low-income country (India) [50], our study found that food insecurity was associated with a significantly lower probability of adherence to physical activity guidelines. More specifically, individuals with food insecurity were 27% less likely to meet the physical activity recommendation, 30% less likely to meet the sedentary behavior recommendation, and 28% less likely to meet both the physical activity and sedentary behavior recommendations compared to those with food security. These results contribute to a growing body of evidence that suggests food insecurity serves as a barrier to health-related behaviors. Although previous research has mostly focused on the dietary consequences of food insecurity [51,52,53], the present study adds to the evidence, explaining that the limitation on obtaining adequate and reliable food may also affect movement behaviors. Food insecurity may hinder physical activity participation though several potential paths. First, those experiencing food insecurity may encounter significant economic challenges, which limit their ability to access recreational facilities, fitness equipment, or structured exercise programs that often require fees or transport [54]. Second, these individuals may have higher levels of stress and psychosocial burden as they have to deal with the problem of scarce food resources [55,56]. Chronic stress can lead to fatigue and weakened motivation, making it more challenging to engage in regular physical activity [57,58].

Regarding sedentary behavior, a previous study found that adults living in households with food insecurity did not have more sedentary time than those living in households without food insecurity [29]. This is inconsistent with our findings, which indicate that individuals with food insecurity were more likely to exceed the recommended sedentary time. This discrepancy may be attributed to the different measurement methods employed. In our work, participants self-reported the total daily sitting time, including leisure-based sedentary activities such as watching television or card playing. The prior study used a physical activity volume threshold of zero to define sedentary behavior, which may not capture the broader range of sitting or reclining behaviors [29]. There are two plausible explanations for the significant relationship between food insecurity and sedentary behavior. First, food insecurity may result in malnutrition, negative emotion, and deteriorating health conditions, which further reduce motivation, decrease physical activity, and potentially increase sedentary behavior. Second, this reduction in physical activity and increase in sedentary behavior can further exacerbate overall health issues, leading to higher medical expenses and decreased productivity, thereby creating a vicious cycle that makes it increasingly difficult for people to afford adequate nutrition.

Furthermore, our findings indicate that individuals in food-insecure environments are less likely to simultaneously meet both physical activity and sedentary behavior recommendations. This dual non-compliance with healthy behaviors is problematic, given that insufficient physical activity combined with extensive sedentary time is associated with a poorer cardiovascular metabolic status and elevated risks of health conditions such as heart disease and diabetes [59]. This may also call for more measures in the future to encourage individuals with food insecurity to engage in physical activities and reduce sedentary behaviors, in order to improve their overall health conditions.

When examining sex-specific variations, our results revealed a stronger and more consistent relationship between food insecurity and movement behaviors among females than males. Females who experienced food insecurity were significantly less likely to meet the physical activity recommendation, the sedentary behavior recommendation, and the combination of both. Conversely, for males, food insecurity was significantly associated with lower adherence to the sedentary behavior recommendation and combined recommendations, but not with physical activity alone. These differences may be attributable to the distinct roles, responsibilities, and social expectations assigned to females in LMICs. Females are often the primary caregivers and frequently manage household duties such as food preparation and looking after children. The stress of these roles, coupled with concerns over limited food resources, may restrict the time and energy available for promoting healthy behaviors, including leisure-time physical activities [60]. Furthermore, the psychological stress of managing household food insecurity may disproportionately affect females, who are often more sensitive to family-level economic and nutritional challenges [61,62]. Females may give up their own health-related activities and instead focus on the immediate nutrition and welfare needs of children or older relatives, effectively reducing their opportunities for exercise or movement breaks throughout the day. On the other hand, males facing food insecurity might still participate in work-related physical tasks, thereby maintaining a certain level of physical activity to some extent. However, the stress of food insecurity may lead to higher sedentary leisure habits, explaining why males who experienced food insecurity were less likely to adhere to the sedentary behavior recommendation, though their compliance with physical activity has not significantly declined.

The findings of this study have important implications for public health policy and interventions. First, food insecurity not only limits dietary quality but also constrains the ability to engage in healthy movement behaviors, creating a vicious cycle of poor health outcomes. Measures designed to increase physical activity or reduce sedentary time among middle-aged and older adults in LMICs should explicitly consider the context of food insecurity. For instance, a food assistance program could integrate a comprehensive module designed to promote regular physical activity. This module may include educational resources on low-cost exercise options and short activity breaks, demonstration classes, as well as the development of a safe and accessible community to facilitate walking or group fitness classes. Second, our findings highlight the importance of implementing gender-specific intervention measures. Given that females seem to be particularly affected by the interaction between food insecurity and movement behaviors, policies should focus on addressing the common issues among females. Possible measures could start from considering how to alleviate the burden on families and caregivers by enhancing social support systems, thereby encouraging females to allocate more time and resources to promoting healthy behaviors.

Despite the valuable insights offered by this study, several limitations should be acknowledged. While this study provides valuable insights, several limitations should be acknowledged. First, the cross-sectional design precludes causal inference. It is uncertain whether food insecurity leads to reduced adherence to movement behavior recommendations or whether individuals with lower adherence are more likely to experience food insecurity due to associated health and economic challenges. Longitudinal studies are needed to establish causal pathways and explore potential bidirectional relationships. Second, the self-reported measures of physical activity and sedentary behavior are susceptible to recall and social desirability biases, which may result in errors in values. Future studies should consider using objective measures, such as accelerometers, to verify and enhance the accuracy of self-reported data. Third, while the models adjusted for several covariates, including age, education, marital status, residential area, and chronic diseases, unmeasured confounders (e.g., access to community resources, social support, and mental health status) may have influenced the results. These factors warrant further investigation in future research.

Additionally, this study has several strengths. The large sample size and inclusion of participants from different LMICs enhances the contribution of these research findings to the current research field. Moreover, the stratified analysis provides an important understanding of gender-specific differences, which are often overlooked in research on food insecurity and movement behaviors. Finally, the adjustment of several key demographic and health-related factors strengthens the validity of the observed associations.

## 5. Conclusions

The results of the current study extend prior research by offering a comprehensive investigation of food insecurity, physical activity, and sedentary behavior among middle and older adults in LMICs using a nationally representative sample. The findings reveal that food insecurity was significantly associated with lower adherence to both physical activity and sedentary behavior recommendations among middle-aged and older adults in LMICs. In these settings, females with food insecurity showed more consistent and significant associations than males. Future longitudinal studies should be conducted to systematically examine whether an enhancement in food security causes continuous changes in movement behaviors.

## Figures and Tables

**Table 1:** Sample characteristics (*n* = 16,454).

Variables		*n*	Mean ± SD/%
Age		16,454	65.47 ± 8.18
Gender	Male	8350	50.75
	Female	8104	49.25
Education (years)	0	3251	19.76
	1–6	7295	44.34
	7–12	4092	24.87
	>12	1816	11.04
Marital status	Yes	11,676	70.96
	No	4778	29.04
Residential areas	Rural	6440	39.14
	Urban	10,014	60.86
Alcohol consumption	Yes	8894	54.05
	No	7560	45.95
Smoking	Never	10,511	63.88
	Current	4272	25.96
	Past	1671	10.16
Number of chronic diseases	0	6499	39.50
	1–2	7674	46.64
	≥3	2281	13.86
Food insecurity	Yes	1210	7.35
	No	15,244	92.65
Meeting sedentary behavior recommendation		14,510	88.19
Meeting physical activity recommendation		10,463	63.59
Meeting physical activity and sedentary behavior recommendations		9616	58.44

Note: SD, standard deviation.

**Table 2:** The associations between food insecurity, physical activity, and sedentary behavior.

	Meeting Physical Activity Recommendation			Meeting Sedentary Behavior Recommendation			Meeting Physical Activity + Sedentary Behavior Recommendations		
	*p*	OR	95%CI	*p*	OR	95%CI	*p*	OR	95%CI
Non-food insecurity		Reference			Reference		Reference		
Food insecurity	<0.001	0.73	0.64–0.83	<0.001	0.70	0.59–0.83	<0.001	0.72	0.63–0.81

Note: OR odds ratio; CI confidence interval. The models are adjusted for age, gender, education level, marital status, residential areas, alcohol consumption, smoking, and number of chronic diseases.

**Table 3:** The associations between food insecurity, physical activity, and sedentary behavior by sex.

	Meeting Physical Activity Recommendation			Meeting Sedentary Behavior Recommendation			Meeting Physical Activity and Sedentary Behavior Recommendations		
	*p*	OR	95%CI	*p*	OR	95%CI	*p*	OR	95%CI
Male									
Non-food insecurity	Reference			Reference			Reference		
Food insecurity	0.15	0.88	0.73–1.05	<0.001	0.61	0.48–0.78	<0.05	0.81	0.68–0.96
Female									
Non-food insecurity	Reference			Reference			Reference		
Food insecurity	<0.001	0.61	0.51–0.73	<0.05	0.77	0.61–0.98	<0.001	0.63	0.53–0.75

Note: OR odds ratio; CI confidence interval. The models are adjusted for age, education years, marital status, residential areas, alcohol consumption, smoking status, and number of chronic diseases.

## Data Availability

The datasets for this study can be requested from the authors.
